# Artificial Intelligence for Diagnosis and Treatment of Dysphagia

**DOI:** 10.1055/s-0044-1801781

**Published:** 2025-01-23

**Authors:** Geraldo Pereira Jotz, Arthur Viana Jotz, Daniel Arnold, Wyllians Vendramini Borelli

**Affiliations:** 1 Universidade Federal do Rio Grande do Sul (UFRGS), Porto Alegre, Brazil; 2 Universidade do Vale do Sinos (UNISINOS), Brazil; 3 Voice Institute, Porto Alegre, Brazil; 4 Pontifícia Universidade Católica do Rio Grande do Sul (PUCRS), Brazil; 5 Memory Center, Hospital Moinhos de Vento (HMV), Brazil

## Introduction

In recent years, technological advances in the use of artificial intelligence (AI) in medicine have demonstrated promising potential in the detection and care of dysphagic patients. Several studies have explored different AI methodologies in the search for diagnostic and therapeutic accuracy for dysphagic patients, while its clinical implementation remains in progress. Improvements in early diagnosis in a scalable manner help public health managers to take more urgent initial measures, always seeking to improve the patient's clinical condition and, mainly, their well-being when it comes to quality of life. Despite promising results in research settings, the transition to widespread clinical use faces important barriers ahead. These include the need for extensive validation in diverse patient populations, integration with existing healthcare systems, and addressing concerns related to data privacy and security. Additionally, there is a need for standardized protocols and guidelines to ensure consistent and reliable use of AI tools in clinical practice. As the field continues to evolve, ongoing collaboration between researchers, clinicians, and technology developers will be crucial to overcoming these challenges and fully realizing the potential of AI in dysphagia management.

In this editorial, we raise the potential use of AI in the diagnosis and treatment of dysphagia. A few selected studies that hold promise for the clinical implementation of AI are discussed, as well as their limitations and further steps. Regarding diagnosis, AI may assist in identifying radiation-free alternatives, remote monitoring, and deep learning methods. Regarding treatment, AI-based treatment is still in its early days with treatment planning.

## AI-assisted Diagnosis for Dysphagia

The benefits of AI-assisted diagnosis for dysphagia include improved accuracy and efficiency, reduced diagnosis time, and the potential for remote diagnosis and monitoring. 1 2 These benefits could lead to earlier interventions and improved outcomes for patients with dysphagia. However, some challenges need to be addressed before AI-assisted diagnosis for dysphagia can be widely adopted, such as high-quality datasets 3 and validation focused on clinical settings.

AI-assisted diagnosis for dysphagia can potentially address some limitations of traditional diagnostic methods such as videofluoroscopic swallowing study (VFSS), fiberoptic endoscopic evaluation of swallowing (FEES), and others. While considered gold standards, these methods can be time-consuming, expensive, require specialized equipment, and pose risks like radiation exposure. 4 5 AI algorithms can be used to analyze images and videos from these procedures to automatically detect signs of dysphagia, potentially leading to earlier and more accurate diagnoses. 6 7

Several studies have demonstrated the potential of AI-assisted diagnosis for dysphagia. AI-assisted was developed for a computer-aided diagnosis (CAD) system called FEES-CAD that analyzes FEES videos to detect aspiration and penetration with high accuracy, comparable to experienced laryngologists. 6 Another study proposed a web application that uses AI to analyze VFSS videos and diagnose dysphagia, classifying it as penetration or aspiration, which can help clinicians recommend appropriate dietary options for patients. 7 Deep learning models have been developed to analyze various types of data, such as voice recordings and swallowing accelerometry signals, for dysphagia screening and prediction. 3 5 8

## AI-based Treatment for Dysphagia

AI-powered treatment strategies show promise for improving dysphagia rehabilitation outcomes. Researchers are exploring several ways to use AI in treatment planning. AI algorithms analyze patient data—including medical history, diagnostic results, and treatment responses—to create individualized therapy plans. This personalized approach could improve treatment effectiveness and patient adherence. 8 9 Studies have also demonstrated the value of real-time feedback during swallowing exercises, which helps patients adjust their techniques and monitor progress. This interactive approach enhances motivation and supports motor learning. 5 While evidence supporting AI in dysphagia treatment continues to grow, clinical implementation remains in its early stages.

## Conclusion

Despite these challenges, AI-assisted diagnosis and treatment for dysphagia show great promise for improving patient care. While further research and development are needed to realize this technology's full potential, future studies should prioritize real-world applications of machine learning models for commonly diagnosed pathologies. Key priorities include refining these models to enhance their accuracy and reliability in clinical settings, particularly in handling diverse datasets and providing real-time analysis. Interdisciplinary collaboration has become increasingly vital in developing and implementing AI tools for dysphagia management. Partnerships between technologists, clinicians, and researchers help ensure that AI applications remain clinically relevant and user-friendly. These collaborations are essential for bridging the gap between technological innovation and practical clinical application.

In sum, beyond diagnosis and treatment, AI applications are expanding into patient monitoring, rehabilitation, and education. These solutions provide comprehensive care that addresses dysphagia's complex nature. As AI technologies advance, we can expect to see more innovative applications spanning the entire spectrum of dysphagia management.
